# High Performance of Functionalized Graphene Hydrogels Using Ethylenediamine for Supercapacitor Applications

**DOI:** 10.3389/fchem.2022.854666

**Published:** 2022-05-17

**Authors:** Hong Ju, Weihui Xu, Lu Fang, Jinzhuo Duan

**Affiliations:** School of Materials Science and Engineering, China University of Petroleum, Qingdao, China

**Keywords:** supercapacitors, functionalized, graphene hydrogel, partial reduction, ethylenediamine

## Abstract

High-performance supercapacitor (SC) electrodes typically require excellent rate capabilities, long cycle life, and high energy densities. In this work, ethylenediamine (EDA) functionalized graphene hydrogels (FGHs) with a high capacitor performance were prepared from graphene oxide (GO) dispersions using a two-step hydrothermal method. In addition, we used a very small amount of EDA to achieve the partial reduction and functional modification of GO, and the synthesized FGH-4 binder-free electrodes exhibited a high specific capacitance of −240 F/g at 1 A/g. We also successfully fabricated a symmetric SC device based on the FGH-4 electrode, with a wide voltage window of 3.0 V. More importantly, the as-assembled symmetric SC delivered a high specific energy of 39 Wh/kg at a specific power of 749 W/kg, while still maintaining its superior cycle life (retaining 88.09% of its initial capacitance after 10,000 cycles).

## Introduction

Since the discovery of graphene in 2004, graphene and its derivatives have received extensive attention as electrode materials for supercapacitors (SCs) due to their excellent chemical and physical properties (Huang et al., 2012; Chabot et al., 2014; Wang et al., 2018; Le et al., 2019). Studies have found that a single graphene layer with a specific area of 2,630 m^2^/g can store a large amount of positive and negative charges (Lin et al., 2015). The theoretical specific capacitance of single-layer graphene as an electrode material with a fully utilized surface area in electric double-layer capacitors can reach 550 F/g (El-Kady et al., 2012). However, the strong Van der Waals interactions between adjacent graphene sheets greatly increase the propensity for the re-stacking or aggregation of graphene sheets during fabrication, which limits their use in SCs (Zhou et al., 2015; Li et al., 2016).

To solve the issue of graphene sheet stacking, scientists have self-assembled 2D graphene sheets into 3D skeletal structures, known as graphene hydrogels and aerogels (Kou et al., 2015; De et al., 2017; Cui et al., 2021). These 3D graphene have an interconnected porous network structure with a high specific surface area, which provides an effective ion transport pathway (Kim et al., 2013). In addition, the 3D graphene materials with a self-supporting structure have excellent mechanical strength and electronic conductivity, and their incorporation in SCs has been successfully realized (Kou et al., 2015; De et al., 2017; Cui et al., 2021).

To tailor its electronic properties, the chemical composition of graphene can be adjusted by dropping graphene with foreign atoms (Duan et al., 2015). Nitrogen is a promising dopant because it has a similar electron number and comparable atomic radius to carbon (Tang et al., 2013). However, current approaches for doping graphene with nitrogen typically require a large dopant quantities and high temperatures, and these methods are time-consuming (Dai et al., 2018). Studies have shown that the fabricated SCs, based on N-doped graphene electrodes, exhibit a relatively low energy densities, which hinders their large-scale practical applications (Liu et al., 2016; Xia et al., 2017). Graphene oxide (GO) contains many oxygen-containing functional groups, which can react with amino containing organic compounds successfully dope nitrogen atoms into graphene (Zhang et al., 2016). Stress defects caused by the doping of nitrogen atoms can also distort graphene sheets, further increasing the specific surface area of graphene (Zhang et al., 2014).

In this work, we presented a facile two-step hydrothermal method to successfully dope nitrogen atoms into graphene sheets using GO as the precursor and ethylenediamine (EDA) as the ammonia source. The graphene sheets subsequently self-assembled into hydrogels. The as-prepared functionalized graphene hydrogels (FGHs) served as binder-free electrode, delivering a gravimetric capacitance of −240 F/g at 1 A/g. Thus, we reported our findings for the design and fabrication of a symmetrical SC device with a wide voltage window of 3.0 V using EMIMBF_4_ as the electrolyte. The device demonstrated a high specific energy (39 Wh/kg at a specific power of 749 W/kg) and excellent cycling stability (88.09% capacitance retention after 10,000 cycles). Furthermore, the device was comparable to lead-acid batteries (35–40 Wh/kg), demonstrating its promising application for use in energy storage systems.

## Experimental Section

### Preparation of GO

GO was prepared using the modified Hummers’ method (You et al., 2013). First, 2 g of graphite powder and 1 g of sodium nitrate were mixed and added to 80 ml of concentrated H_2_SO_4_ at 10°C. Then, we slowly added 12 g of KMnO_4_ to the mixed solution over 2 h, ensuring that the temperature of the solution did not exceed 10°C. After 2 h, the beaker containing the mixed solution was removed and placed in a constant temperature water bath at 35°C for 30 min, after which it was poured into 160 ml of distilled (DI) water for dilution. After 15 min, 500 ml of 75°C DL water and 30 ml of H_2_O_2_ were added to the beaker in turn, and the mixture was stirred for 3 h. Afterward, the mixture was filtered and washed with a dilute HCl aqueous solution (200 ml) to remove the metal ions, then the mixture was washed with DI water wash several times until the solution reached a pH value of 7. Finally, the product was collected and freeze-dried.

### Preparation of FGHs

Forty milligrams of GO were added to 20 ml of DI water yielding a yellow-brown solution, and followed by 1 h of ultra-sonication. Using a hydrothermal process, the FGHs were prepared with the GO solution and EDA. For FGH-3, 30 μl of EDA and 20 ml of the GO dispersion were mixed in a beaker, stirred vigorously for 30 min, and then ultra-sonicated for 30 min. The resulting solution was sealed in a 25 ml Teflon lined autoclave for 3 h at a constant at 90°C, then rapidly heated to 180°C for 12 h. After naturally cooling to room temperature, the sample was rinsed with deionized water until it reached a pH of 7, and then was allowed to air dry. Samples FGH-4, FGH-5, and FGH-6 were denoted by their volume of EDA. For comparison, graphene hydrogel (GH) was obtained using a similar method without the addition of EDA into the aqueous GO dispersion. Table 1 lists the sample details.

**TABLE 1:** Names of the samples.

Samples	The quality of GO (mg)	The volume of EDA (μl)
GH	40	0
FGH-3	40	30
FGH-4	40	40
FGH-5	40	50
FGH-6	40	60

### Sample Characterization

Sample phase structures were analyzed using X-ray diffraction (XRD) (D8 advanced diffractometer with Cu K_α_ radiation), and Raman spectra were obtained using a DXR Raman microscope with a 532 nm laser. Fourier transform infrared (FT-IR) spectra were recorded under a Nicolet6700 spectrometer with a wave number range of 400–4,000 cm^−1^. TGA tests were recorded on a Q600 SDT thermal analyzer under nitrogen protection, between 20 and 800°C. The electronic states of the surface elements were characterized using X-ray photoelectron spectroscopy (XPS) (ESCALAB 250Xi electron spectrometer with Al K_α_ radiation). The specific surface area was obtained using the Brunauer–Emmett–Teller (BET) method. Sample microstructures were observed using scanning electron microscopy (SEM) (JEOL JSM-7200F).

Methylene blue (MB) adsorption is the standard method for measuring the specific surface area of graphitic materials, where 1 mg of adsorbed MB molecules will cover a surface area of 2.54 m^2^ (Xu et al., 2013). Therefore, the MB dye adsorption method was employed to measure the specific surface areas of the FGHs and GH. The surface areas were calculated by adding the FGHs or GH into 20 ml of MB solution (0.04 mg/ml) for 7 days to achieve the adsorption equilibrium. Then the MB concentration was determined by analyzing the supernatant through UV-vis spectroscopy at a wavelength of 665 nm, after which we compared it to the initial concentration of MB before interacting with FGHs or GH.

### Electrochemical Measurements

The electrochemical performance of the FGHs and GH was measured using a symmetrical three-electrode system. A slice of wet FGHs or GH was cut from the cylindrical samples and wrapped with filtrate paper to remove excess water. Afterward, the slice was pressed onto Pt foil at 10 MPa for 30 s and used as the working electrode (1 cm diameter), leading to an active material loading of 3–4 mg/cm^2^, which was soaked in electrolyte for 12 h before electrochemical tests were conducted. The counter and reference electrodes were used with the Pt foil and standard Ag/AgCl electrode, respectively, and 1.0 M H_2_SO_4_ aqueous solution was used as the electrolyte.

The electrochemical measurements of the SC were carried out to evaluate the FGH-4 films for practical applications in symmetric coin cells using a two-electrode system in the EMIMBF_4_ electrolyte. FGH-4 slices were first immersed in pure ionic liquid EMIMBF_4_ under a vacuum at 100°C for 24 h. Residual water residual in the electrodes was removed by vacuum evaporation, and then the electrodes were transferred to 1.0 M EMIMBF_4_ electrolyte in acetonitrile (AN) solution for another 24 h (Pan et al., 2018). Then, two of the same FGH-4 films were used directly as electrodes, separated by a glass fiber membrane, and soaked with the EMIMBF_4_ electrolyte. All of the components were assembled into coin cells in an Ar filled glove box.

All electrochemical experiments were carried out using the ModuLab electrochemical workstation. Electrochemical impedance spectroscopy (EIS) measurements were performed using a frequency range from 0.01 Hz to 100 kHz at an amplitude of 5 mV. Cyclic voltammetry (CV) and galvanostatic charge/discharge (GCD) tests were performed at different scan rates and current densities. In addition, the life cycle tests were conducted using galvanostatic charge/discharge measurements at a constant current density of 10 A/g for 10,000 cycles. The mass specific capacitances (C_s_), which were derived from the galvanostatic discharge curves, were calculated based on the following equation:

$${∁}_{s}=\frac{I\times \Delta t}{m\times \Delta V}$$ (1)

In addition, the specific energy (E) was estimated by

$$\text{E}=\frac{1}{2}C_{s}\Delta V^{2}$$ (2)

and the specific power (P) was estimated by

$$\text{P}=\frac{E}{\Delta t}$$ (3)

where *I* is the discharge current, Δ*t* is the time to full discharge, *m* is the mass of the electrode, and Δ*V* represents the voltage window after a full charge.

## Results and Discussion

### FCH Synthesis Mechanism Analysis

**SCHEME 1:**
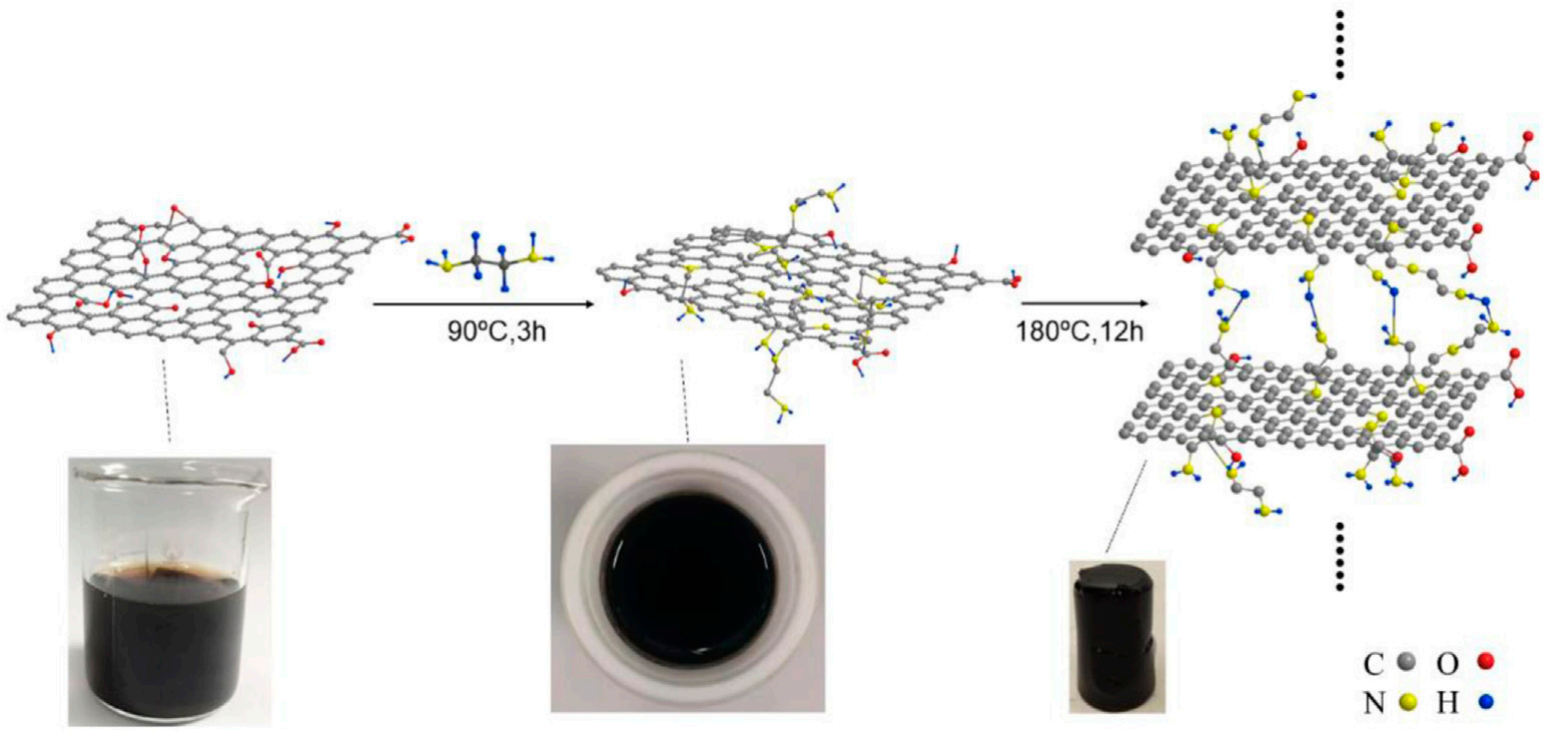
Illustration showing the FGH reaction mechanism.

Scheme 1 shows a sketch of the FGH reaction mechanism. During the first step, EDA acts as an ammonia source, realizing the successful source of amino groups on the graphene sheets, as well as a reduction agent to remove the oxygen functional groups in GO. The second step was carried out at 180°C for 12 h, where high temperature and long-term reactions further removed the oxygen functional groups in GO, and the sp^2^ hybridized graphene network was restored. In addition, the EDA molecular chain was attached to the graphene plane through amino acid reactions with oxygen-containing groups on the GO surface, which inhibited π-π stacking in the single graphene sheet. Other amino acids on the molecular chain generated hydrogen bonds with the amino acids in the other graphene sheet, thus increasing the distance between the graphene sheets. When GO was reduced, the reduced GO sheets became cross-linked with each other due to hydrophobic effects, forming a 3D framework structure with sandwiched water molecules.

### Characterization of the Samples

**FIGURE 1:**
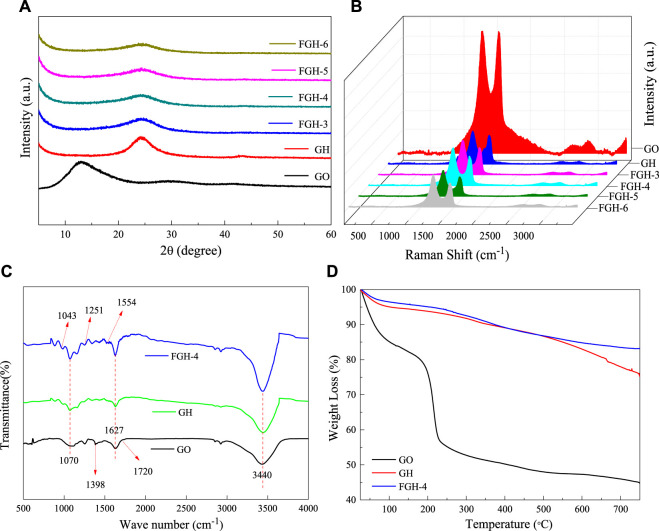
**(A)** XRD, **(B)** Raman, **(C)** FTIR, and **(D)** TGA samples results.

Figure 1A shows the XRD patterns of GO, GH, and the FGHs. For the GO sample, a characteristic diffraction peak appeared at 2θ = 12.95° (002), corresponding to an interlayer distance of 6.83 Å. This was larger than the basal spacing of natural graphite (3.35 Å), indicating complete exfoliation of the graphite. After the reaction, we found a broad diffraction peak centered around 2θ ≈ 24.35° (Table 2) for the GH and FGHs, which was in accordance with the characteristic peak of graphite (Huang et al., 2015). In addition, the characteristic diffraction peak for the FGHs gradually broadened with increasing EDA content, indicating that most of the oxygen-containing groups from GO were removed during the hydrothermal process and the formation of multiple-layers of graphene (Zhang et al., 2014).

**TABLE 2:** XRD and Raman spectral data for the samples.

Samples	2 theta (ο)	d value (Å)	D-band peak (cm^−1^)	G-band peak (cm^−1^)	I_D_/I_G_
Graphite		3.34			
GO	12.95	6.83	1,348.36	1,592.94	1.00
GH	24.22	3.67	1,348.55	1,595.53	1.15
FGH-3	24.30	3.66	1,350.88	1,596.05	1.27
FGH-4	24.42	3.64	1,357.37	1,598.63	1.28
FGH-5	24.40	3.65	1,350.68	1,596.89	1.33
FGH-6	24.39	3.65	1,355.98	1,598.89	1.34

We used Roman spectroscopy analysis to investigate the disordered structural features of the GO, GH, and FGH samples. As depicted in Figure 1B, the G and the D bands of the GH and FGHs shifted to a higher frequency region, compared to GO after the hydrothermal process. This indicated the reduction of GO and doping of N atoms in the graphene sheets. The D band was related to the disorders or defects in the carbon structure, while the G band was ascribed to the ordered graphitic structure. The relative intensity ratio of the D and G peaks (I_D_/I_G_) was denoted by the degree of graphitization, defects, or the domain size of graphitization in the carbon materials (Pimenta et al., 2007). The I_D_/I_G_ value (Table 2) of the FGHs was higher than GH and GO, indicating that the presence of nitrogen atoms on the graphene sheets increased the structural disorder and number of defects in the FGHs.

As shown in Figure 1C, the FTIR spectra of GO fluctuated significantly, indicating numerous oxygen functional groups on the GO sheets obtained by Hummers oxidation. For example, C-O was attributed to the epoxy functional group at 1,070 cm^−1^, C-O in the alcohol or phenol structures was located at 1,398 cm^−1^, and C=O in the carboxylic acid and carbonyl moieties was located at 1,627 and 1720 cm^−1^. These were clearly visible in the FTIR spectrum of GO (Lai et al., 2012). The O-H stretching vibrations due to residual water molecules were also observed at −3,440 cm. The above-mentioned peaks strongly decreased or disappeared in the GH sample, indicating the dehydration and elimination of the oxygen functional groups during the hydrothermal process. For the FGH-4 sample, in addition to the significant attenuation of the corresponding peaks, new peaks appeared at 1,251, 1,043, and 1,554 cm^−1^. These were attributed to C-N and N-H stretching vibrations in the FGH-4 sample (Zhang et al., 2016). In the FGH-4 sample, the peak intensity at around 3,440 cm^−1^ was slightly stronger than GH and GO, and this was attributed to the interactions of N-H and O-H bonds in the FGH-4 sample.

The TGA test characterized the weight loss of samples with temperature, allowing us to evaluate the thermal stability of the samples. As shown in Figure 1D, the TGA curve of GO displayed three significant weight loss stages. The first weight loss stage occurred below 100°C and was attributed to the absorption of water (Lei et al., 2012). The second weight stage loss occurred between 130 and 220°C, and was ascribed to the decomposition of the thermally unstable oxygen functional groups (Zhu et al., 2010). The third weight loss stage occurred above 220°C, and was due to the removal of the more stable oxygen functional groups on the GO sheets as the temperature increased (Jiang et al., 2013). The stability of the GH and FGH-4 samples was higher than GO, indicating that most of the oxygen functional groups were removed during the hydrothermal process. The weight loss of the GH and FGH-4 samples above 500°C was possibly due to the decomposition of the oxygen functional groups such as carbonyl, either, or quinine (Liang et al., 1999; Lei et al., 2012; Zhang et al., 2016). Figure 1D shows that the weight loss of the FGH-4 sample was smaller than the GH sample, indicating that the reduction degree during the hydrothermal process with the addition of EDA was higher than the hydrothermal process without the addition of EDA. These results were also consistent with the Raman and FTIR data.

**FIGURE 2:**
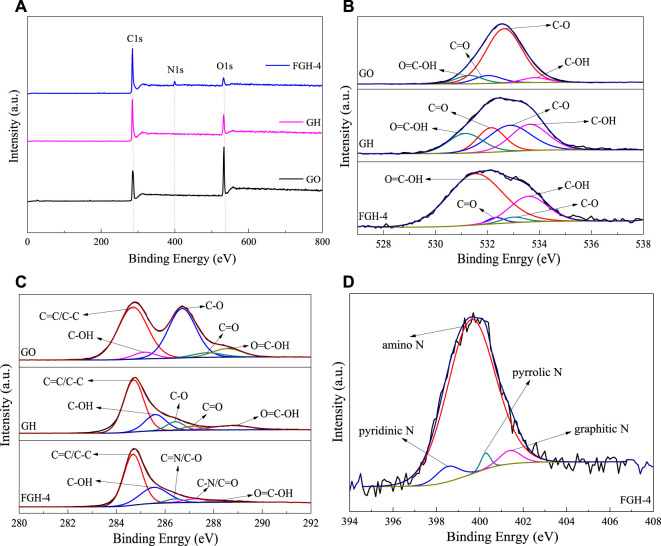
**(A)** XPS survey spectra, **(B,C)** O1s and C1s spectra for GO, GH, and FGH-4, respectively, and **(D)** N1s region spectra for FGH-4.

The chemical states of the elements in the GO, GH, and FGH-4 samples were analyzed by XPS. Figure 2A shows the C1s, O1s, and N1s peaks found in the sample survey spectra. The surface atomic concentrations of C, O, N, and C/O were derived from the corresponding peak areas from XPS, and the results are summarized in Table 3. Numerous oxygen functional groups were successfully removed, and the N atoms were successfully grafted by hydrothermal reduction. Figure 2B shows the high-resolution spectrum of O1s in the three samples, which were fitted to several corresponding peaks, namely O=C-OH (531.33 eV), C=O (532.16 eV), epoxy or alkoxy C-O (532.83 eV), and C-OH (533.68 eV). The deconvolution of the C1s spectra yielded five peaks in each of the three samples, specifically, C=C/C-C (284.69 eV), C-OH (285.43 eV), C=N/C-O (286.51 eV), C-N/C=O (287.37 eV), and O-C=O (288.48 eV), which are shown in Figure 2C. The peaks for C-O and C=O decreased significantly, indicating that GO gradually exhibited a restored structure dominated by C-C bonds. The presence of C=N and C-N peaks demonstrated the incorporation of N into the graphene sheets. Specifically, the N1s peak in the FGH-4 sample Figure 2D was fitted to several peaks corresponding to the pyridinic N (398.52 eV), amino N (399.6 eV), pyrrolic N (400.27 eV), and graphitic N (401.4 eV). Figure 2D shows that the N in the FGH-4 sample was present in the form of the amino N in the graphene sheets, because the amino groups in the EDA tail chain were completely retained. The other amino groups in the EDA molecular chain were attached to the graphene plane through amino reactions with the oxygen-containing groups on the GO surface, namely as pyrrole N, pyridinic N, and graphitic N forms. The high-level distribution of N atoms endowed the FGH-4 electrode with high density active sites and good wettability (Zhang et al., 2017). Pyridinic N and pyrrolic N offer great pseudo-capacitance, and can improve conductivity, resulting in enhanced capacitive performance (Deng et al., 2016; Liu et al., 2016). Graphitic N can also promote interactions with the anions in the electrolyte, forming an electrical double layer, which can enhance SC capacitance (Ornelas et al., 2014; Deng et al., 2016). Amino N also increased the hydrophilicity of the sample, thereby accelerating the ion transport rate of the electrolyte inside the sample. Therefore, FGH-4 would be a good candidate for high-performance SC electrodes.

**TABLE 3:** Elemental content and C/O ratios, as obtained by XPS sample analysis.

Samples	C (at%)	O (at%)	N (at%)	C/O
GO	69.69	30.31		2.30
GH	82.77	17.23		4.80
FGH-4	85.36	8.15	6.49	10.47

**FIGURE 3:**
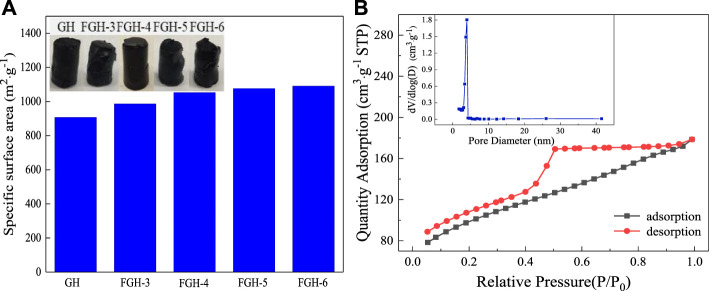
**(A)** The specific surface area histogram of the samples, **(B)** BET adsorption-desorption curve and pore size distribution (inset) for FGH-4.

Because the FGHs were used directly as SC electrodes without freeze-drying, the MB dye adsorption method was employed to determine the intrinsic surface area of the wet FGHs. Figure 3A shows that the specific surface areas of the GH and FGH samples increased with the addition of EDA. However, the macroscopic volume of these samples was approximately the same, possibly because many macropores were produced in the interior of the FGH samples with the increasing addition of EDA. The specific surface area and pore structure of FGH-4 were further investigated by nitrogen adsorption-desorption measurements Figure 3B. FGH-4 also displayed a typical type-IV isotherm with a large hysteresis loop (H2) in the middle pressure range (P/P_0_ = 0.45–0.95), suggesting mesoporous characteristics (Sun et al., 2015; Thangappan et al., 2020). However, the adsorption and desorption curves in the low-pressure range are not completely closed, possibly due to the presence of some micropores. Brunauer–Emmett–Teller (BET) and Barrett–Joyner–Halenda (BJH) (inset in Figure 3B) analyses also revealed that FGH-4 had a high specific surface area of 337.27 m^2^/g, pore volume of 0.21 cm^3^/g and a dominant pore size of 3–4.25 nm. We also observed a few pores that were approximately 26 nm, and smaller than 3 nm, in size. These were attributed to nitrogen doping, which reduced the agglomeration or restacking of the graphene sheets, creating 3D porous structures consisting of graphene sheets with interconnected open pores (Dai et al., 2018). Additionally, the macropores acted as highways for electrolyte transport, and the wide pore size distribution was beneficial for energy storage (Lan et al., 2016; Shao et al., 2016).

**FIGURE 4:**
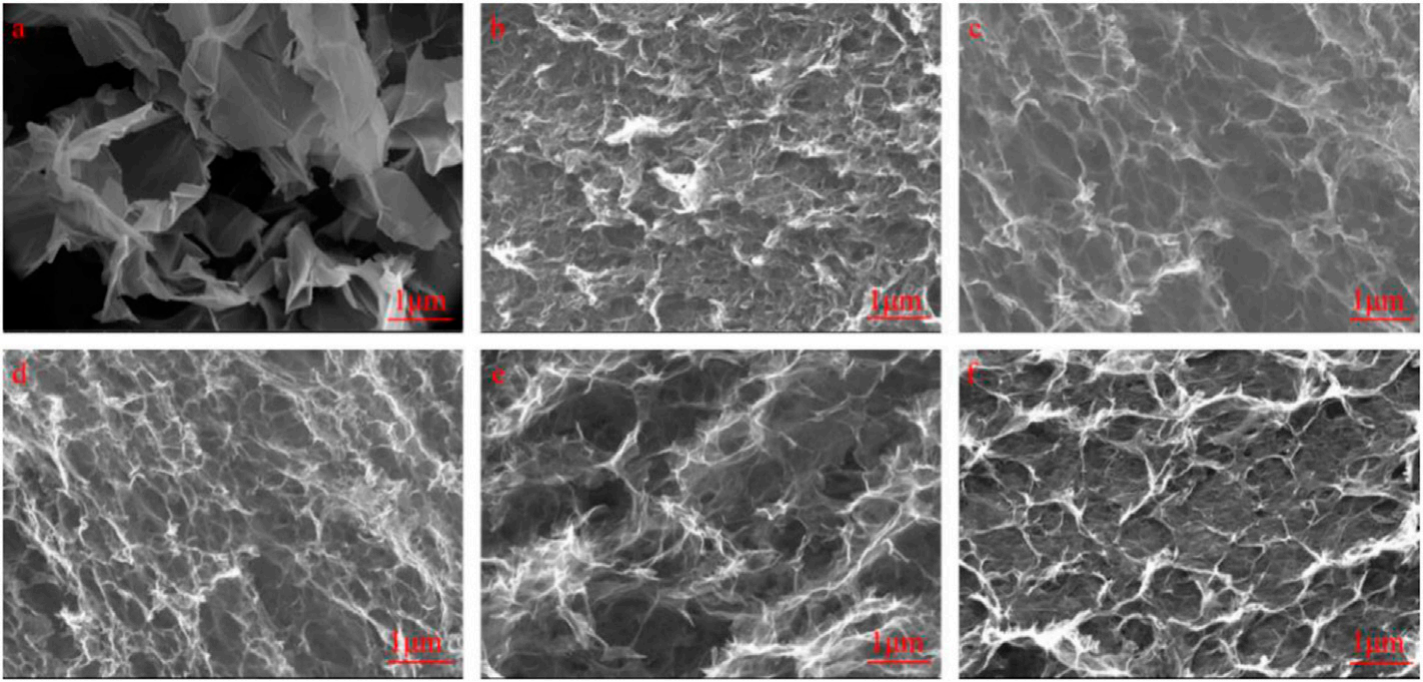
SEM images of **(A)** GO, **(B)** GH, **(C)** FGH-3, **(D)** FGH-4, **(E)** FGH-5, and **(F)** FGH-6.

We used SEM to observe the interior microstructures of GO, GH, and the FGHs after freeze drying. As shown in Figure 4A, the morphology of GO prepared using the Hummers method resembled thin paper, and some GO sheets were closely stacked together. Uneven ruffles were distributed in the middle of the GO sample, which were attributed to the presence of the oxygen functional groups in the GO sheets. Figures 4B–F shows that both GH and FGH samples had an interconnected 3D porous network structure, due to the π-π stacking interactions and hydrophobic effect of the graphene sheets during the hydrothermal process (Rao et al., 2014). Figure 4B shows that some of the GH sheets were stacked together, and pore walls formed. This was possibly caused by an insufficient degree of GH reduction. When EDA reacted with GO, the tail chain of EDA was introduced into the graphene sheets, generating hydrogen bonds. This inhibited π-π stacking in the single graphene sheet and expanded the space between the graphene sheets. With the increasing addition of EDA, the FGH sample becomes flatter, and the pore size gradually increased, which was consistent with the results obtained from the MB dye adsorption method.

### Electrochemical Performance Characterization

**FIGURE 5:**
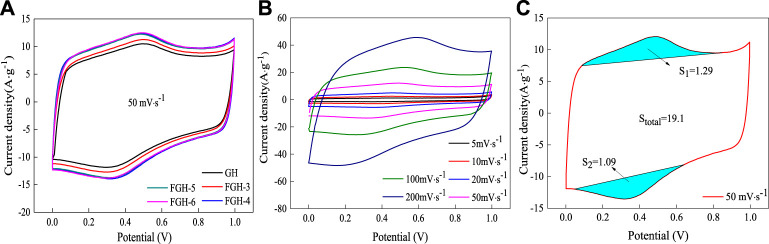
The CV curves for the samples in 1 M H_2_SO_4_: **(A)** different samples at a scan rate of 50 mV/s, **(B)** FGH-4 at different scan rates, and **(C)** the CV split graph of FGH-4 at a scan rate of 50 mV/s.

We first investigated the electrochemical performance of the SC electrode material samples using a 3-electrode configuration with 1 M H_2_SO_4_ aqueous electrolyte. Figure 5A compares the CV curves of the GH and FGH-based electrode materials at a scan rate of 50 mV/s. The CV curves for all samples were approximately rectangular, while the CV curves for the FGH samples had a large area, benefiting from the higher degree of reduction, greater specific surface area, and the nitrogen-containing functional groups on the graphene sheets in the FGHs. The FGH-4 sample required the smallest quantity of EDA, for approximately the same capacitance. For additional study, we tested the CV of the FGH-4 sample at different scan rates ranging from 5 to 200 mV/s. As shown in Figure 5B, the CV curves for the FGH-4 sample remained rectangular for all scan rates, and the CV profile shapes were almost unchanged, while the loop area became larger with increasing scan rates. This indicates that the FGH-4 sample had a quick charge propagation capability and excellent rate performance. Moreover, the CV curves for the FGH-4 sample appeared as distinct humps at higher scan rates, indicating that the rapid Faradaic redox reactions occurred in the surface layer of the FGH-4 sample. FTIR and XPS analyses indicated that some of the nitrogen and oxygen functional groups were distributed inside the FGH-4 sample, and these functional groups reacted with H_2_O in the aqueous electrolyte, which contributed to the Faraday capacitance (Zhang et al., 2016). Figure 5C shows the contribution of Faraday capacitance, based on the CV specific capacitance calculation formula:

$$C_{m}=\frac{\int IdV}{2vmV}$$ (4)

We concluded that the specific capacitance contributed by these functional groups was about 23.8 F/g, which was only 12.5% of the total capacitance of FGH-4. Therefore, the FGH-4 sample mainly exhibited EDLC behavior.

**FIGURE 6:**
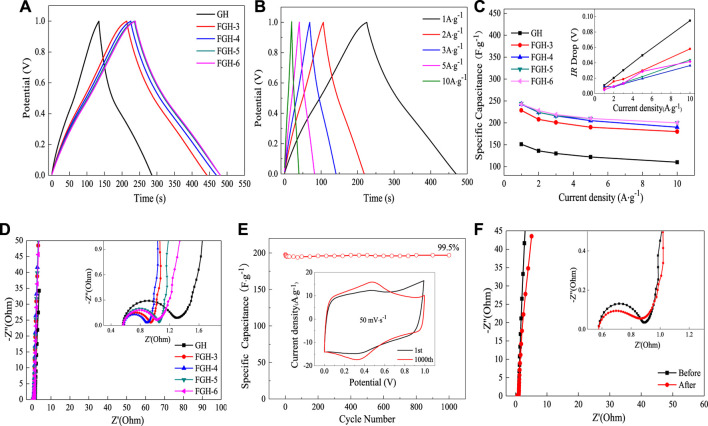
**(A)** GCD curves of the different samples at a current density of 1 A/g, **(B)** GCD curves of FGH-4 at different current densities, **(C)** gravimetric specific capacitance and IR drop curves (inset) of different samples at different current densities, **(D)** EIS curves of different samples in 1 M H_2_SO_4_, **(E)** cycle performance and CV split graph at a scan rate of 50 mV/s (inset) for FGH-4, and **(F)** EIS curves of FGH-4 before and after 1,000 cycles.

Figure 6A compares the charge/discharge curves of the GH- and FGH-based electrode materials at a constant current density of 1 A/g. The FGH samples had longer charge and discharge times compared to the GH sample at the same current density, revealing that FGH-based electrode materials could store more energy than GH. In addition, we observed the GCD curves of the FGH-4 sample at different current densities. All curves were in the form of typical isosceles triangles, and highly linear, as shown in Figure 6B, revealing that the FGH-4 SC electrode material exhibited excellent capacitive characteristics. Figure 6C shows that the FGHs exhibited higher capacitance than GH, for all current densities examined in this study. The specific capacitances of FGH-4 were calculated as 242.8, 224, 216, 205, and 190 F/g at current densities of 1, 2, 3, 5, and 10 A/g, respectively. However, the specific capacitances of GH were only 151.2, 136.2, 129.9, 122, and 110 F/g, respectively, at the same current densities. In addition, the IR drop of the FGH samples (inset in Figure 6C was smaller than GH, especially for the FGH-4 sample, indicating that the FGH-based electrode materials had a smaller internal resistance compared to GH. Therefore, the FGH-4-based SC electrode material exhibited a larger capacitance and better rate capacitance compared to GH.

EIS can further evaluate the facilitated ion-transport kinetics and electrode conductivity of GH and the FGHs. Figure 6D shows that the Nyquist plots of the GH and FGHs samples in the low-frequency region were nearly parallel to the *Y* axis, which was attributed to the small diffusive resistance between the electrolyte and electrode materials, revealing fast ion diffusion and ideal capacitive behavior. The semicircles in the high-frequency region represented the charge-transfer resistance in the electrode materials, and the first intersection of the semicircle with the real axis corresponded to the equivalent series resistance (ESR). As shown in the inset in Figure 6D, FGH-4 had a smaller semicircle and ESR than the other samples. The slope of the 45° section of the curve in the high-frequency region represented the Warburg resistance, indicating frequency-dependent ion diffusion in the electrolyte-electrode interfaces. In the inset in Figure 6D, the Warburg line for FGH-4 was shorter compared to the other samples. This was attributed to the presence of nitrogen-containing functional groups in the graphene sheets, the modified macroporous 3D framework, and the higher specific surface of FGH-4, which promoted faster diffusion of the electrolyte ions inside the network structure.

A long cycling life is a critical factor for the practical applications of SCs. Figure 6E shows the specific capacitance retention of FGH-4, as evaluated by the GCD technique between 0 and 1 V at a high current density of 10 A/g for 1,000 cycles. FGH-4 still maintained a capacitance of 99.5% after 1,000 charge/discharge cycles, indicating that FGH-4 had outstanding cycle stability. The inset in Figure 6E shows that the FGH-4 CV curves at a scan rate of 50 mV/s were nearly perfect rectangles before and after 1,000 cycles, while the CV curve after 1,000 cycles contained symmetric redox peaks, and the pattern area further increased. This was ascribed to the gradual activation of the electrode material and increased effective interfacial area between the electrode material and the electrolyte with increased reaction time (Yoon et al., 2013). For further study, we tested the EIS curve of FGH-4 after 1,000 cycles Figure 6F. Compared to the EIS curve before 1,000 cycles, the ESR of FGH-4 was slightly reduced, and the Warburg line was also shorter, indicating that the electrolyte ions were in full contact with FGH-4, resulting in a shorter ion diffusion path for FGH-4.

**FIGURE 7:**
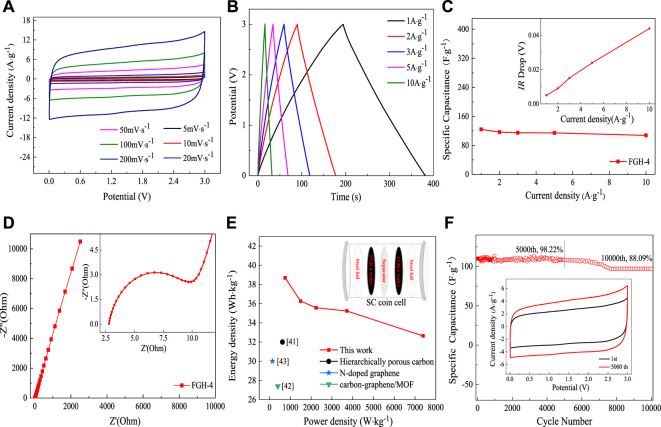
**(A)** CV curves for the FGH-4 coin cell at different scan rates, **(B)** GCD curves of the FGH-4 coin cell at different current densities, **(C)** the gravimetric specific capacitance and the IR drop curves (inset) of the FGH-4 coin cell at different current densities, **(D)** EIS curves of the FGH-4 coin cell, **(E)** Ragone plots of the FGH-4 coin cell, and **(F)** cycling performance of the FGH-4 coin cell.

To better illustrate the practical performance of the FGH-4 material, we constructed a symmetrical coin cell using two pieces of FGH-4 with the same size and weight, and without any other additives. We then tested these in the BMIMBF_4_ electrolyte. Figure 7A shows the CV curves of the FGH-4 coin cell at different scan rates within a voltage window of 0–3.0 V. All CV curves retained nearly rectangular shapes, even at a high scan rate of 200 mV/s, further verifying the excellent capacitive behavior of the FGH-4 electrode. GCD curves for the coin cell were measured at different current densities, as shown in Figure 7B. The nearly symmetrical triangular shape of the GCD curves further implied the excellent capacitive behavior of the FGH-4 coin cell. At room temperature, the FGH-4 coin cell electrode in the BMIMBF_4_ electrolyte delivered a specific capacitance of up to 124.2 F/g at a current density of 1 A/g Figure 7C, which was approximately consistent with the aqueous electrolyte. Even at 10 A/g, the coin cell still retained a gravimetric capacitance of 107.6 F/g with a small voltage (IR) drop of 0.04 V (inset in Figure 7C). The excellent performance was due to the high electrical conductivity and fast ion diffusion of the FGH-4 electrode, which was reflected by the lower resistance, as shown in Figure 7D. Energy and power density are two important parameters for evaluating the electrochemical behavior of SCs (Dai et al., 2017). As shown in the Ragone plot in Figure 6E, the coin cell delivered high specific energy of 38.69 Wh/kg at a specific power of 748.75 W/kg. Even at a high specific power of 7390 W/kg, it still retained a specific energy of 32.64 Wh/kg. The obtained specific energy densities were much higher than previously reported symmetrical SCs based on similar electrode materials, including hierarchically porous carbon (32 Wh/kg at a specific power of 620 W/kg) (Cheng et al., 2015), carbon-graphene/MOF (30 Wh/kg at a specific power of 137 W/kg) (Meng et al., 2016), and N-doped graphene (27.4 Wh/kg at a specific power of 400 W/kg) (Hao et al., 2015).

The cycle life of SCs is another important parameter that can be used to evaluate capacitance for practical applications (Pan et al., 2018). The FGH-4 coin cell electrode in the BMIMBF_4_ electrolyte exhibited cycling stability with a capacitance retention rate of 98.22% after 5,000 charge/discharge cycles at a high current density of 10 A/g (Figure 7F), and 88.09% of the capacitance was maintained even after 10,000 cycles. This also demonstrated the promising potential of this material for practical applications. Moreover, the CV loop area after 5,000 cycles (inset in Figure 7F) became larger, also indicating that the FGH-4 coin cell electrode had quick charge propagation capability and excellent rate performance.

## Conclusion

Utilizing EDA as an ammonia source and a reduction agent, we used a facile two-step hydrothermal reaction method to successfully achieve the partial reduction and functional modification of GO. Using this method, we safely and successfully achieved the self-assembly of GO into 3D macroscopic graphene hydrogels. The results showed that the nitrogen atoms in the FGH samples were successfully grafted into the graphene sheets. As a result of the modified macroporous 3D framework, the presence of oxygen and nitrogen functional groups, and the high specific surface, the FGH-4 coin cell electrode exhibited excellent capacitance behavior and cycle stability. This material also displayed a wide voltage window, and high specific energy in the BMIMBF4 electrolyte. Therefore, based upon these advantages, the prepared FGH-4 material has promising potential as an electrode material for SCs.

## Data Availability

The original contributions presented in the study are included in the article/Supplementary Material, further inquiries can be directed to the corresponding author.
